# Genome-wide association studies of yield-related traits in high-latitude japonica rice

**DOI:** 10.1186/s12863-021-00995-y

**Published:** 2021-10-05

**Authors:** Guomin Zhang, Rongsheng Wang, Juntao Ma, Hongru Gao, Lingwei Deng, Nanbo Wang, Yongli Wang, Jun Zhang, Kun Li, Wei Zhang, Fengchen Mu, Hui Liu, Ying Wang

**Affiliations:** 1 Biotechnology Research Institute, Heilongjiang Academy of Agricultural Sciences, Harbin, China; 2 Northern Japonica Rice Molecular Breeding Joint Research Center, Chinese Academy of Sciences, Harbin, China

**Keywords:** *Oryza sativa japonica*, GWAS, Yield trait, Resequencing, Rice breeding

## Abstract

**Background:**

Heilongjiang Province is a high-quality japonica rice cultivation area in China. One in ten bowls of Chinese rice is produced here. Increasing yield is one of the main aims of rice production in this area. However, yield is a complex quantitative trait composed of many factors. The purpose of this study was to determine how many genetic loci are associated with yield-related traits. Genome-wide association studies (GWAS) were performed on 450 accessions collected from northeast Asia, including Russia, Korea, Japan and Heilongjiang Province of China. These accessions consist of elite varieties and landraces introduced into Heilongjiang Province decade ago.

**Results:**

After resequencing of the 450 accessions, 189,019 single nucleotide polymorphisms (SNPs) were used for association studies by two different models, a general linear model (GLM) and a mixed linear model (MLM), examining four traits: days to heading (DH), plant height (PH), panicle weight (PW) and tiller number (TI). Over 25 SNPs were found to be associated with each trait. Among them, 22 SNPs were selected to identify candidate genes, and 2, 8, 1 and 11 SNPs were found to be located in 3′ UTR region, intron region, coding region and intergenic region, respectively.

**Conclusions:**

All SNPs detected in this research may become candidates for further fine mapping and may be used in the molecular breeding of high-latitude rice.

**Supplementary Information:**

The online version contains supplementary material available at 10.1186/s12863-021-00995-y.

## Background

Rice cultivated in Asia is the staple food for most of the population worldwide. Research on its genetic variation, population structure and diversity has advanced greatly in recent decades [1–3]. Cultivated rice belongs to different subspecies or varietal groups and shows different domestication characteristics. Additionally, the domesticated subspecies include two main groups*: Oryza sativa japonica* and *O. sativa indica*. However, evidence suggests that they may have been domesticated separately from the ancestral species approximately 18 and 12 thousand years ago [4]. Genomic studies have confirmed the differentiation of three subspecies within *O. sativa japonica*, temperate, subtropical and tropical japonica, which grow in diverse environments with different climate characteristics [5].

Every subspecies may have distinctive signatures or alleles that are formed during domestication or artificial selection. People in a specific area selected particular traits for their consumption needs [6]. Many studies of different genes showed clear evidence of positive selection during the evolutionary process, such as genes related to waxiness and cold tolerance [7–9]. Research focused on a subspecies or a population collected from a specific geographical region may reveal distinctive characteristics. Moreover, functional alleles or loci will be identified with certain analysis methods.

Quantitative trait locus (QTL) analysis has turned out to be a very effective tool for gene and locus discovery in recent years. A large number of genes have been cloned based on QTLs from different species around the world [10–12]. The emergence of high-throughput genome sequencing technology has decreased the expense and enhanced its efficiency. Combined with population phenotypes, many statistical analysis measures have been developed based on next-generation sequencing or single-nucleotide polymorphism (SNP) chips. This approach is called genome-wide association study (GWAS) and became widely used within a short time after being proposed. Its three main advantages over other population analysis methods are higher mapping resolution, a larger allele number and broader reference population, and lower time consumption [13]. Genome-wide association studies can be performed in a wide range of populations, such as germplasm resource material, [14, 15] F2 populations, [16] nested association mapping (NAM) populations, [17] multiple advanced generation inter-cross (MAGIC) populations [18, 19] and random open-parent association mapping (ROAM) populations [20, 21]. Multiple statistical models can be used in GWAS based on different populations or scales of SNP numbers, [22, 23] and population structure and genetic relationships can be taken into consideration [24, 25]. It has been more than 10 years since GWAS was first proposed, [26] and many mature workflows and analysis tools have been developed [27, 28]. The application of GWAS in rice has been widely reported in recent years. Alleles or SNPs located by GWAS have been applied in rice molecular breeding [29, 30].

Yield is one of the main traits that rice breeders focus on because of its relevance to worldwide food security. Nearly half of the world population consumes rice as a staple food. Yield is also known to be a multigene controlled trait, and many genes and loci have been found that could account for yield differentiation. Furthermore, yield is a complicated trait that is affected by many other traits, such as the tiller number, plant height, grain number, grain weight and number of primary branches [31, 32]. Association analysis with different populations may identify some unique genes or loci that contribute to specific traits. Therefore, it is necessary to identify novel genes or loci in a different population that may play a role only in a specific environment.

Rice cultivated at high latitudes in Asia shows many good characteristics, such as cold tolerance and high quality. There might be a large number of effective alleles that would be useful for further breeding for these traits. Few studies have focused on high-latitude natural populations and their effective alleles. In this research, we collected hundreds of cultivars and landraces as a natural population from high-latitude areas, including Northeast China, South Korea, Russia and Japan, and performed GWASs to examine their PH, PW, TI and DH in four different environments in Heilongjiang Province, China, with the aim of discovering genes or associated SNPs that could account for the differentiation of phenotypes and are expected to be used in further breeding programs.

## Results

### Phenotyping of different traits

**Fig. 1:**
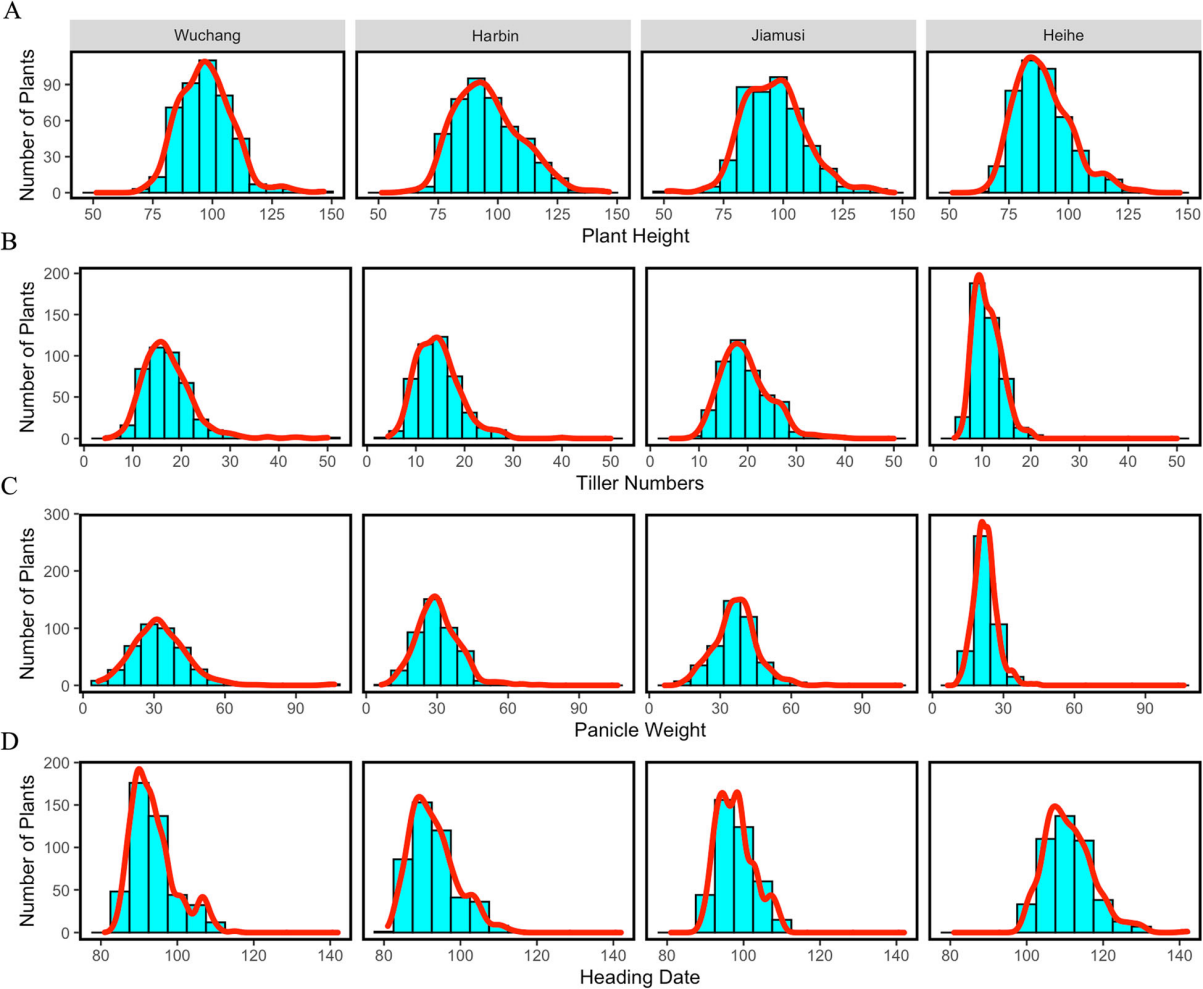
Histogram of 450 plants in Wuchang, Haerbin, Jiamusi and Heihe (from left to right of each line): PH (**A**), TI (**B**), PW (**C**), DH (**D**). The red line indicates the fitted curve of the distribution

The plant height of 450 accessions ranged from 51.33 cm to 146.67 cm, with an average of 93.67 cm (Supplementary Table 2). In all four locations, plant height showed a normal distribution and a similar median with no significant difference except in Heihe, which may be due to a short growing season (Fig. 1A). The TI and PW in the four locations showed differences from each other, and in Heihe, these two traits showed narrower ranges than in the other locations (Fig. 1B-C). The DH showed a gradient change with increasing latitude. Wuchang and Harbin have similar means of DH, 94 and 93, respectively. However, at higher latitudes, DH significantly increased because of the longer daylight (Fig. 1D). Analysis of variance for the four traits showed significant differences (< 0.001). However, broad-sense heritability gave a higher estimate for PH (0.89) and DH (0.95) and a lower estimate for TI (0.68) and PW (0.47).

### Resequencing results and SNP distribution

Approximately 1227 billion bases in total were obtained after resequencing, with an average of 6.3× sequencing depth and 18.9 million reads for each accession (Supplementary Table 1). Over 6.4 million SNP loci were called among the 450 accessions, indicating an average of 57 bp between pairs of SNPs. SNPs on each chromosome ranged from 0.39 million (chromosome 3) to 0.76 million (chromosome 11), with an average of 0.54 million per chromosome. According to the minor allele frequency (MAF) statistics, nearly 65% had an MAF less than 0.05, 20.6% had an MAF greater than 0.1 and 8.1% had an MAF greater than 0.25. The mean MAF on each chromosome varied between 0.052 and 0.085, with an average of 0.07 across all chromosomes.

### Linkage disequilibrium and SNP distribution

**Fig. 2:**
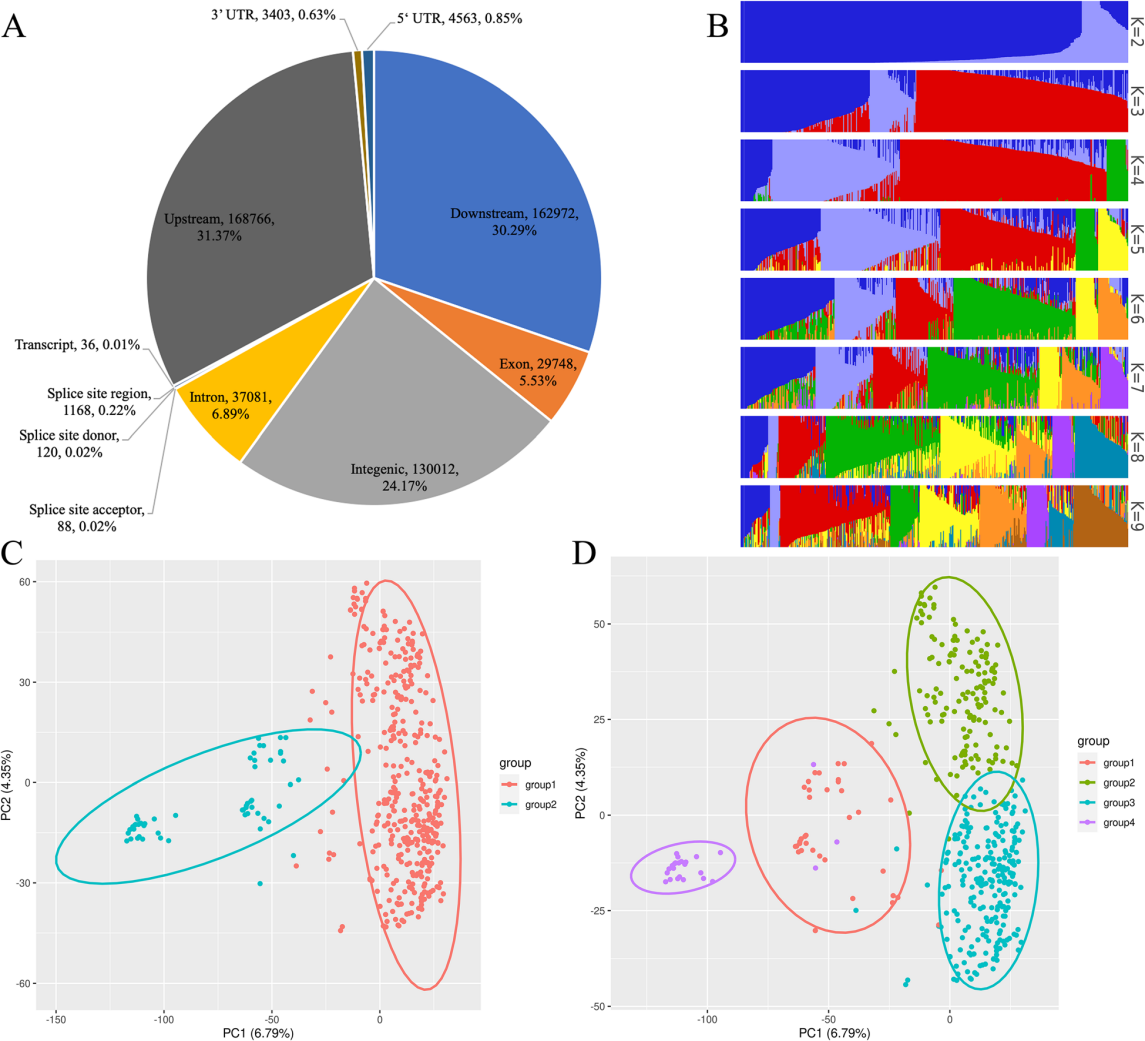
**A**. Number of effects of all SNPs by genomic region. **B** Population structure for different K values. Each accession is represented by a vertical bar, and each colour indicates accessions belonging to one subgroup. **C.** PCA plots of the first two components of 450 accessions divided into two groups. **D**. PCA plots of the first two components of 450 accessions divided into four groups

After filtering by MAF and missing genotype rate, 1,991,545 SNPs remained, and all these SNPs were used for linkage disequilibrium (LD) decay analysis across all chromosomes. The LD decay distance ranged from 15 kb to 27 kb, with an average of 20 kb in the four groups predicted by population structure analysis (Supplementary Fig. 1A). However, the LD decay distance of all accessions was 23 kb, which means that *r*^*2*^ dropped to half of its maximum value (Supplementary Fig. 1B). This LD decay distance was lower than the previous estimate in temperate japonica but higher than that in *O. rufipogon,* [33] which may be affected by landraces in the population and possibly by having undergone weakened artificial selection. Finally, we filtered the SNPs based on the *r*^*2*^ value, and 189,019 SNPs were retained for subsequent analyses, indicating one SNP every 2 kb across the whole genome. These SNPs corresponded to 194,313 variants and 537,957 effects. Most SNPs were downstream of genes (30.29%), intergenic (24.17%) or upstream of genes (31.37%) (Fig. 2A).

### Genetic diversity and population structure

According to the population structure evaluation, when K = 2, all 450 accessions were divided into two groups. Group 1 contained 396 accessions (88.0%), mostly breeding varieties. Group 2 contained 54 (12.0%) accessions, mostly cultivar introductions (Fig. 2B). When K = 3, the Group 1 subdivided into two groups, but Group 2 had no change. When K = 4, the two groups subdivided from Group 1 maintained with a few individuals changed, but Group 2 subdivided into two groups. For K larger than 4, the two groups subdivided from Group 2 maintained, but the Group 1 subdivided into more groups (Supplementary Table 1).

To further illustrate the population structure of our research panel, principal component analysis (PCA) was performed. When the first and second eigenvectors were used, all accessions could be divided with four subgroups (Fig. 2C, Supplementary Fig. 1A). However, when divided into more than four groups, Group 2 was divided into more than two groups that showed indistinct boundaries (Fig. 2D, Supplementary Fig. 2B-F). Based on the above results, it is more representative when all accessions were divided into four groups and suitable for further analysis. But greater difference were shown between Group 1 and Group 2 when K = 2.

**Fig. 3:**
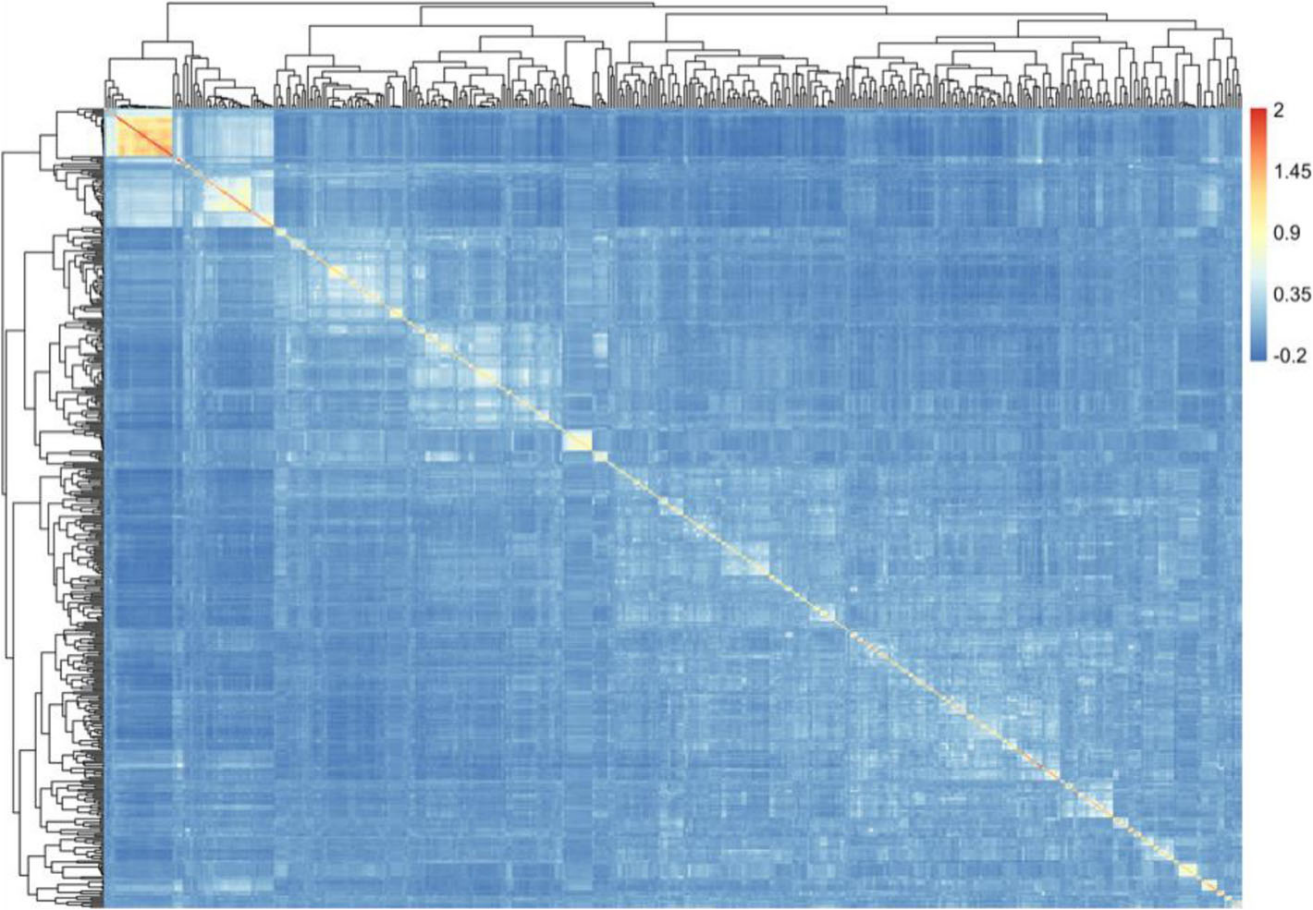
Heatmap of genetic relationships. The colour change from cyan to red indicates an increase in the kinship value from − 0.2 to 2

A kinship matrix was calculated to detect the genetic relationship within the population. The coefficient of relatedness ranged from − 0.25 to 2.01, and a kinship heat map was drawn to visualize the relationships. It is clear that only the upper left corner has a relatively close relationship, and the other accessions have a lower coefficient of relatedness (Fig. 3), indicating that the population used in our research conform to a natural populations but with few relatedness between some accessions.

### Genome-wide association analysis

**Fig. 4:**
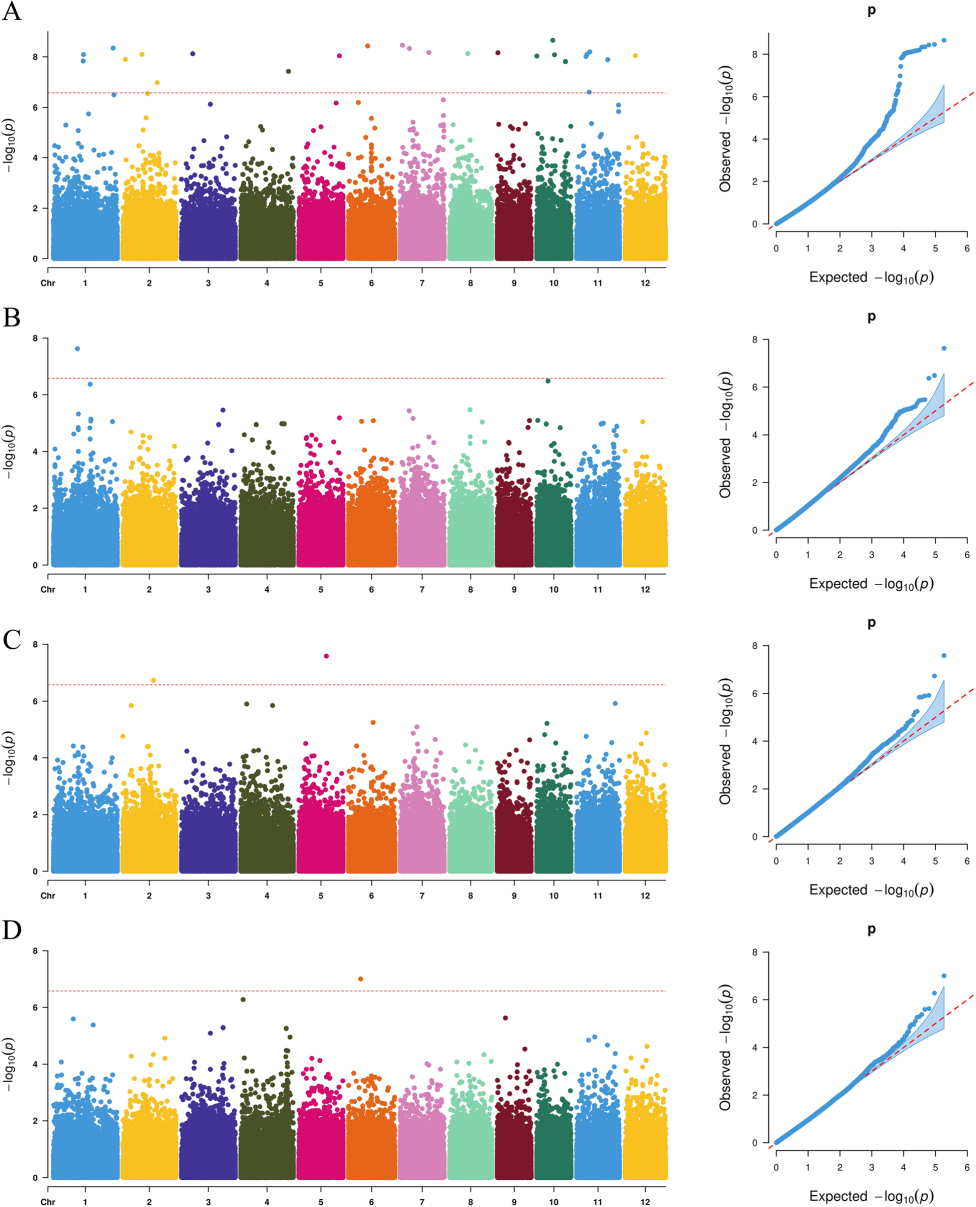
Manhattan plots and QQ plots for the four traits in Heihe by MLM. **A**. Days to heading. **B**. Plant height. **C**. Panicle weight. **D**. Tiller number

The GLM found 597 SNPs to be significantly associated with four traits in total, but the MLM found only 322 (Table 1, Supplementary Table 3). For DH traits, no identical locus was found between the two models. By GLM, 144 loci were significantly associated with DH, and 25 loci were found at more than two locations on chromosomes 4, 6, 7 and 11. However, by MLM, only 2 and 25 SNPs were found to be significantly associated with DH in Wuchang and Heihe (Table 1, Fig. 4A, Supplementary Fig. 5A). Twenty-five of them throughout all 12 chromosomes were from Heihe. Three SNPs on chromosome 7 shows larger effects and detected in multi-environment (Table 2). For PH, 49 SNPs were found in three locations by GLM, 14, 6 and 29 SNPs in Harbin, Heihe and Wuchang, respectively, 2 of which on chromosome 11 and chromosome 12 were detected in both Heihe and Wuchang. Twenty-five SNPs were identified in only two locations by MLM, 1 and 24 in Heihe and Wuchang (Table 1, Fig. 4B, Supplementary Fig. 3B, Supplementary Fig. 5B). Twenty-three were detected by different methods at the same time or the same locations. Only 1 SNPs shows larger effects than other positions on chromosome 5 (Table 2). For TI, 53, 51, 9 and 3 SNPs were found by GLM in Harbin, Wuchang, Jiamusi and Heihe, respectively. By MLM, 41 and 2 were found in Wuchang and Harbin, but in Jiamusi and Heihe, only one for each location. Interestingly, all SNPs detected by MLM in Harbin, Jiamusi and Wuchang were also detected by GLM, but no SNPs were detected between any two locations by the same method (Table 1, Fig. 4D, Supplementary Fig. 3-5D). Among these SNPs, 8 were larger effects and detected by two models. For PW, the most significantly associated loci, 247 and 225, were found by GLM and MLM, respectively. By GLM, only 1 and 4 SNPs were detected in Harbin and Heihe, respectively, but 242 were detected in Wuchang across all 12 chromosomes. Similar to GLM, MLM detected only 1 and 2 SNPs in Harbin and Heihe but 222 in Wuchang (Fig. 4C). Notably, all SNPs detected by MLM were also detected by GLM, except for 2 SNPs in Wuchang (Table 1, Supplementary Fig. 3-5C). Among these SNPs, 8 were larger effects and detected by two models. Interestingly, 3 SNPs (S4_32507995, S5_2003327, S11_8842451) were detected for both TI and PW by GLM (Table 2).

**Table 1:** Summary of significantly associated loci identified by different methods

	GLM				MLM			
	HA	WU	JA	HE	HA	WU	JA	HE
PH	14	29	–	6	–	24	–	1
PW	1	242	–	4	1	222	–	2
TI	53	51	9	3	2	41	1	1
DH	8	83	16	78	–	2	–	25

**Table 2:** Twenty larger effect SNPs and three pleiotropic SNPs

Trait	Model	Location	Marker	Chr^a^	Position	LOD^b^
DH	GLM	HA/HE/WU	S7_8832791	7	8,832,791	8.2062/13.9051/11.5954
DH	GLM	HA/HE/WU/JA	S7_29566846	7	29,566,846	9.1926/14.0176/10.8388/9.5634
DH	GLM	HA/HE/WU/JA	S7_29238189	7	29,238,189	8.7844/14.7984/11.7642/8.9488
PH	GLM/MLM	WU	S5_11687290	5	11,687,290	8.5651/8.1938
TI	GLM/MLM	WU	S2_9226198	2	9,226,198	11.5279/10.4231
TI	GLM/MLM	WU	S2_13626408	2	13,626,408	9.3376/8.1539
TI	GLM/MLM	WU	S2_34946356	2	34,946,356	11.4379/10.0110
TI	GLM/MLM	WU	S4_7050530	4	7,050,530	14.1317/11.9763
TI	GLM/MLM	WU	S7_3214984	7	3,214,984	10.1969/8.6595
TI	GLM/MLM	WU	S9_8477108	9	8,477,108	10.5361/9.0133
TI	GLM/MLM	WU	S11_8842451	11	8,842,451	8.4670/8.1336
TI	GLM/MLM	WU	S11_26127237	11	26,127,237	9.8739/8.1161
PW	GLM/MLM	WU	S1_29521211	1	29,521,211	17.7022/14.8049
PW	GLM/MLM	WU	S3_13365688	3	13,365,688	10.2207/9.2723
PW	GLM/MLM	WU	S4_16690715	4	16,690,715	10.0706/9.1917
PW	GLM/MLM	WU	S4_22584137	4	22,584,137	10.7148/8.4903
PW	GLM/MLM	WU	S6_8044409	6	8,044,409	10.6151/8.7926
PW	GLM/MLM	WU	S7_9482292	7	9,482,292	9.7741/8.7687
PW	GLM/MLM	WU	S8_22791456	8	22,791,456	16.3799/14.3599
PW	GLM/MLM	WU	S11_5366552	11	5,366,552	10.1661/8.6426
TI/PW	GLM	WU	S4_32507995	4	32,507,995	9.0813/8.4376
TI/PW	GLM	WU	S5_2003327	5	2,003,327	7.2883/8.5209
TI/PW	GLM	WU	S11_8842451	11	8,842,451	8.4670/8.1495

^a^Chromosome

LOD value in different locations or with different models

### Candidate gene identification

A total of 317 SNPs detected either by two models or in more than two locations were selected for candidate gene identification. Of them, 223 SNPs were used for PW, and 19 missense variants, 1 splice region variant and 1 stop gained locus were identified (Table 3). Four missense variants were identified for TI, 3 for PH and only 1 for DH. Among these SNPs, Candidate genes of twenty two larger effect or pleiotropic SNPs were identified. Within gene regions, eleven SNPs were found. Two of them were 3 prime UTR variants, eight were found to be intron variants and only one SNP was synonymous variant. Eleven SNPs were located in intergenic region. The distance between SNPs and the nearest genes ranged from 0 to 18.9 kb (Table 4). All these genes execute unknown biology functions in *O. sativa japonica* group, according to the Rice Genome Annotation Project database.

**Table 3:** Summary of annotation variants for four traits

Traits	DH	PH	PW	TI
number of SNPs	25	25	223	44
number of variants	27	26	229	46
number of effects	81	64	589	133
chromosome	4, 6, 7, 11	1, 3–8, 10–12	1–12	1–12
3′ UTR variant	–	–	3	–
5′ UTR variant	2	–	1	–
downstream gene variant	29	19	170	38
frameshift variant	–	–	1	–
intergenic region	16	18	143	31
intron variant	11	2	33	10
missense variant	1	3	19	4
splice region variant	–	–	1	–
stop gained	–	–	1	–
synonymous variant	–	3	31	4
upstream gene variant	22	19	187	46

**Table 4:** Candidate genes identified from 22 detected SNPs for 4 traits

SNP	Chr^a^	Gene ID^b^	Annotation	Distance (kb)^c^
S1_29521211	1	LOC_Os01g51330	3 prime UTR variant	0.0
S2_9226198	2	LOC_Os02g16230	intergenic region	2.3
S2_13626408	2	LOC_Os02g22820	intergenic region	18.9
S2_34946356	2	LOC_Os02g57080	intron variant	0.0
S3_13365688	3	LOC_Os03g23110	intron variant	0.0
S4_7050530	4	LOC_Os04g12744	intergenic region	2.5
S4_16690715	4	LOC_Os04g28234	3 prime UTR variant	0.0
S4_22584137	4	LOC_Os04g37960	intron variant	0.0
S4_32507995	4	LOC_Os04g54690	intergenic region	0.0^d^
S5_11687290	5	LOC_Os05g19990	synonymous variant	0.0
S5_2003327	5	LOC_Os05g04370	intergenic region	0.5
S6_8044409	6	LOC_Os06g14406	intron variant	0.0
S7_8832791	7	LOC_Os07g15330	intron variant	0.0
S7_29566846	7	LOC_Os07g49370	intron variant	0.0
S7_29238189	7	LOC_Os07g48870	intergenic region	3.9
S7_3214984	7	LOC_Os07g06610	intergenic region	0.9
S7_9482292	7	LOC_Os07g16240	intergenic region	3.4
S8_22791456	8	LOC_Os08g36160	intergenic region	2.5
S9_8477108	9	LOC_Os09g14350	intron variant	0.0
S11_8842451	11	LOC_Os11g15590	intron variant	0.0
S11_26127237	11	LOC_Os11g43300	intergenic region	3.3
S11_5366552	11	LOC_Os11g10010	intergenic region	0.9

^a^Chromosome

^b^Gene ID from MSU genome annotation database (version 7.0)

^c^The distance between SNPs and the nearest gene. The 0 kb means that the SNP located inside the gene

^d^The distance between the SNP S4_32507995 and the gene LOC_Os04g54690 is 28 bp

## Discussion

Many models have been reported for use in GWAS, [23, 34, 35] and GLM and MLM are two that have been used frequently to analyse a variety of plants [15, 36]. In GLM, the principal components or population structure needs to be taken into consideration as a fixed effect. However, in MLM, relative kinship should be added as a random effect, although the result is still less efficient for large data sets. Many other algorithms have been developed to address this problem, such as the compressed MLM, [35] efficient mixed-model association expedited (EMMAX) algorithm and [37] and factored spectrally transformed linear mixed model (FaST-LMM) [38]. When different models are compared, some of them show high statistical power but low computational speed, while others show intermediate statistical power but very fast computational speed [26]. For further gene screening, these models should be adapted to increase the accuracy of the associations and narrow down the possible associated interval.

Association analysis was first used on populations of unrelated human individuals, [39] but it is difficult to collect natural plant populations with distant genetic relationships in a local area. Although many accessions collected in this research were from South Korea, Russia and Japan, many of them were elite varieties derived from the same ancestral parents. Meanwhile, information on some cultivars was lost, resulting in unknown origins. Genetic population analysis also showed that the distinctions among some of these accessions were obscure (Fig. 3), so further research is needed to optimize the population structure and screen the research panels to obtain a clearer population structure [40, 41].

Principal component analysis has been shown to be a substitute for population structure in GWAS [42]. Therefore, we chose the first five components as the population structure matrix to conduct a GWAS. The eigenvalue derived from PCA was proportionally low because of the large population scale (6.79% for the first principal component, data not shown). The smooth downward trend of the eigenvalue made it difficult to choose the number of components for association analysis (Supplementary Fig. 6 ) [43]. The different ways of dividing population groups by population structure and PCA eigenvector also made it difficult to select a population structure matrix. Therefore, more population structure matrices may be needed for further analysis to locate the key associated SNPs.

A total of 144 DH-related SNPs were detected in four locations by GLM, and 25 of them were detected in more than two locations, which implied that even at different latitudes, heading date was functionally affected by the same genes. Little attention has been given to the associations of PW in cereal crops, but relationships with grain yield and rice quality have been reported [44, 45]. The PW is also used as a main trait for association analysis in rice [46]. However, in our study, too many loci were associated with PW across all 12 chromosomes (Fig. 4C, Supplementary Fig. 3-5C). Therefore, it was not easy to identify the true related genes among these SNPs. More association analysis models may be needed to narrow down the candidate genes. In addition, PW is a comprehensive trait consisting of many factors, such as panicle length, number of grains, and grain weight, which adds to the difficulties of detecting associated sites. A separate analysis of this trait may be needed for further association studies.

From the Manhattan plots, it is obvious that DH shows significant peak values by GLM: 2 peak values on chromosome 2, 1 on chromosome 4 and 2 on chromosome 7 (Supplementary Fig. 7). The peak value of chromosome 4 was located at 14,818,439 bp in the 5′ UTR of the LOC_Os04g25560 gene, which is referred to as the OsSCP23 (putative serine carboxypeptidase homologue) gene. The two peak values of chromosome 7 located at 8,832,791 bp and 29,566,846 bp corresponded to the intron variant LOC_Os07g15330 and the intron variant LOC_Os07g49370, respectively. Both putative genes have unclear functions. Notably, these two genes were located on chromosome 7, close to two reported heading date genes, Ghd7 (LOC_Os07g15770) and DTH7 (LOC_Os0749460), which are approximately 320 kb and 50 kb in length, respectively [47, 48]. In chromosome 2, the location of the peak value is far from the reported genes LOC_Os02g39710 and LOC_Os02g49230 [49, 50]. It may be concluded that many factors can cause false positive results in GWAS, so a wider screening range is needed to choose the affected genes around the associated SNPs. LD blocks will also provide a reference criterion for the range. Various issues need to be considered for further gene screening.

## Conclusion

In this study, 450 accessions were used to perform whole-genome resequencing, and 189,019 SNPs were used for GWAS after filtering according to the MAF, missing genotype rate and *r*^*2*^ value. Bonferroni correction was used to set the threshold of significantly associated SNPs to -Log_10_(P) ≥ 6.58. In total, 597 and 322 significantly associated loci were detected for the four traits by GLM and MLM, respectively. After filtered, 22 larger effect or pleiotropic SNPs for the four traits were used to identify candidate genes. Eleven SNPs were identified within coding regions, two of them were located in 3′ UTR, eight in intron region and one in coding region. The rest of 11 SNPs were found to be located in intergenic region.

All these candidate genes associated with the four yield traits could be used for further gene identification or fine mapping, and related SNPs will also provide guidance for rice breeding in high-latitude areas.

## Materials and methods

### Plant materials

A collection of 450 temperate japonica rice varieties was used as a GWAS panel, including landraces and cultivars collected from Japan, North Korea, Russia, Heilongjiang Province, Jilin Province and Liaoning Province in China, and other unknown origins (Supplementary Table 1). Many landraces and foreign varieties have been introduced into Heilongjiang Province of China in recent decades. Moreover, a number of intermediate varieties were added to the panel for further analyses.

### Field cultivation and management

All materials were planted in the field in 2015. Four experimental fields were located at Wuchang (44.9°N, 127.2°E), Harbin (45.8°N, 126.6°E), Jiamusi (46.8°N, 130.4°E) and Heihe (50.2°N, 127.5°E) in Heilongjiang Province, and all these locations were used as paddy fields for successive years. Wuchang is the most fertile black land of China, with approximately 145 days above the minimal temperature. At higher latitudes, Harbin and Jiamusi have fewer days above the minimal temperature. Heihe is not only the highest latitude of the Chinese temperate zone but also the highest latitude of the world where rice is cultivated. The growth season in Heihe is less than 120 days. Experiments were constructed with a complete randomized design. Ten plants of each accession were used for a single row with 13 cm spacing within the plants and 30 cm spacing between the rows. Field management was conducted normally for a local paddy field.

### Phenotypic evaluations and data statistics

The PH was evaluated before harvest and was measured from ground to the highest panicle tip. The TI was counted in each plant with panicles. The main panicle in each plant was collected and weighed from the rachis internode to obtain the PW. The mean of three plants was calculated as the final value. The heading date was noted when over 50% of plants in a row were heading, and the number of days from sowing to heading was used as DH for further analysis. Phenotype statistics and distribution analyses were performed with the R/base package. Analyses of variance for the four traits were performed using the lmerTest package in R by Student’s t test with a confidence level of α < 0.001 (https://cran.r-project.org/web/packages/lmerTest/index.html). Lines and locations were treated as random effects, and traits were treated as fixed effects with the formula Trait ~ (1|line) + (1|location) by lme4/R (https://github.com/lme4/lme4). The broad-sense heritability (*H*^*2*^) of the four traits was calculated by the following equation: H^2^ = Vg/(Vg + Ve/L), where Vg is the variance of genotypes, Ve is the variance of environments, and L is the number of locations. Statistical plots were drawn with ggplot2/R (http://had.co.nz/ggplot2/).

### Genome resequencing and genotype filtering

Young leaves were collected from each accession, and genomic deoxyribonucleic acid (DNA) was isolated by a rapid method to obtain high-quality total DNA (DOI:10.21769/BioProtoc.1010106). Paired-end libraries were constructed and sequenced on an Illumina HiSeq sequencing system (Illumina, USA). The Nipponbare genome (MSU version 7.0) was used as a reference genome. All reads were aligned to the reference genome with the Burrows-Wheeler Alignment (BWA) tool [51]. After the alignment, quality control was performed with SAMtools (Ver. 1.7), [52] and the Genome Analysis Toolkit was used for SNP calling (GATK, v3.4–46). The UnifieldGenotyper of GATK was used for multiple SNP calling [53]. Genotype imputation was performed using Tassel (Version 5.0) with the LD KNNi Imputation plugin [27].

### Minor allele frequency and linkage disequilibrium

The MAF was calculated with Plink (Version 1.9, http://www.cog-genomics.org/plink2/). After genotype imputation, SNPs were filtered by Plink with thresholds of MAF greater than 0.05 and a missing genotype rate greater than 0.2. Whole-genome LD decay was estimated by pairwise squared correlation coefficients (*r*^*2*^) between SNPs in PopLDdecay [54]. The pairwise *r*^*2*^ value was calculated when all accessions were divided into four groups by population genetic analysis. The LD decay distances were determined where the average *r*^*2*^ dropped to half of its maximum value. The SNPs were further filtered according to the *r*^*2*^ value in Plink with parameters --indep-pairwise 50 10 0.2.

### Population genetic analyses

Population structure analysis, including group estimation, best K value selection and population structure plotting, was performed with fastSTRUCTURE (version 1.0) by the structure, chooseK and distruct plugins, respectively [55]. The PCA and kinship matrix were calculated within TASSEL (Version 5.0) [27]. The first five components were used for further association analysis. The PCA plots of the first two components were drawn with different groups predicted from K values. The kinship heatmap was drawn with pheatmap/R (Version 1.0.12, https://cran.r-project.org/web/packages/pheatmap/index.html). The clustering distance of rows and columns used correlation as its parameter, and the complete parameter was used for the clustering method.

### Genome-wide association analysis

The GWAS analysis was carried out with TASSEL 5, and GLM and MLM were used to detect significantly associated loci. After filtering by LD value, 189,019 SNPs were used for association analysis with a threshold LOD value of 6.58. Genotypes and phenotypes were used in the GLM model with the first five components of PCA as the population structure matrix. However, in the MLM, a kinship matrix was also added as a relatedness covariation. The threshold values for associated SNPs were obtained by the Bonferroni correction, which was calculated as follows: -Log_10_(P) ≥ −Log_10_(0.05/189,019) ≈ 6.58. Manhattan plots and QQ plots were drawn by CMplot/R (Version 3.6.2, https://cran.r-project.org/web/packages/CMplot/).

### Candidate gene identification

Loci that were detected in more than two locations or by both methods were used for candidate gene identification. The Nipponbare genome (MSU version 7.0, http://rice.plantbiology.msu.edu/) annotation database was used as a reference database. SnpEff (Version 4.3 T) was used to annotate significantly associated SNPs [56].

## Supplementary Information

**Additional file 1: Supplementary Table 1.** List of 450 accessions with there resequencing results and grouping information.

**Additional file 2: Supplementary Table 2.** Phenotypes of all accessions in four locations.

**Additional file 3: Supplementary Table 3.** List of all significant postitions detected by two models with 4 locations.

**Additional file 4: Supplementary Fig. 1.** (A) Linkage disequilibrium of all accessions across 12 chromosomes. (B) Linkage disequilibrium differences between four groups predicted by population structure analysis when K = 4. **Supplementary Fig. 2** Plots of the first two principal components in PCA with data points divided into 3 (A) and 5 to 9 (B-F) groups by faststructure. **Supplementary Fig. 3.** Manhattan plots and QQ plots for the four traits in Harbin by MLM. (A) Days to heading. (B) Plant height. (C) Panicle weight. (D) Tiller number. **Supplementary Fig. 4.** Manhattan plots and QQ plots for the four traits in Jiamusi by MLM. (A) Days to heading. (B) Plant height. (C) Panicle weight. (D) Tiller number. **Supplementary Fig. 5.** Manhattan plots and QQ plots for the four traits in Wuchang by MLM. (A) Days to heading. (B) Plant height. (C) Panicle weight. (D) Tiller number. **Supplementary Fig. 6.** Line plot of eigenvalues with the first 20 principal components. A significant downward trend was shown for the first 5 principal components. **Supplementary Fig. 7.** Manhattan plots and QQ plots for Days to heading in four locations by GLM. (A) Heihe. (B) Jiamusi. (C) Harbin. (D) Wuchang.

## Data Availability

Raw reads of 450 accessions used in this study were a part of BioProject PRJCA000322 in the National Genomics Data Center (https://ngdc.cncb.ac.cn) and can be accessed by accession ID listed in Supplementary Table 1.

## Abbreviations

GWAS: Genome-wide association studies
GLM: General linear model
MLM: Mixed linear model
DH: Days to heading
PH: Plant height
PW: Panicle weight
TI: Tiller number
SNP: Single-nucleotide polymorphism
DNA: Deoxyribonucleic acid
QTL: Quantitative trait locus
MSU: Michigan State University
MAF: Minor allele frequency
BWA: Burrows-Wheeler Alignment
LD: Linkage disequilibrium
PCA: Principal component analysis
LOD: Likelihood of odds
UTR: Untranslated region
