# Supplementary material for: Genome-wide association studies of yield-related traits in high-latitude japonica rice
Source: BMC Genom Data. 2021 Oct 5;22:39. doi: 10.1186/s12863-021-00995-y (PMC8493688; doi:10.1186/s12863-021-00995-y)
Supplement: Supplementary file 4 — Additional file 4: Supplementary Fig. 1. (A) Linkage disequilibrium of all accessions across 12 chromosomes. (B) Linkage disequilibrium differences between four groups predicted by population structure analysis when K = 4. Supplementary Fig. 2 Plots of the first two principal components in PCA with data points divided into 3 (A) and 5 to 9 (B-F) groups by faststructure. Supplementary Fig. 3. Manhattan plots and QQ plots for the four traits in Harbin by MLM. (A) Days to heading. (B) Plant height. (C) Panicle weight. (D) Tiller number. Supplementary Fig. 4. Manhattan plots and QQ plots for the four traits in Jiamusi by MLM. (A) Days to heading. (B) Plant height. (C) Panicle weight. (D) Tiller number. Supplementary Fig. 5. Manhattan plots and QQ plots for the four traits in Wuchang by MLM. (A) Days to heading. (B) Plant height. (C) Panicle weight. (D) Tiller number. Supplementary Fig. 6. Line plot of eigenvalues with the first 20 principal components. A significant downward trend was shown for the first 5 principal components. Supplementary Fig. 7. Manhattan plots and QQ plots for Days to heading in four locations by GLM. (A) Heihe. (B) Jiamusi. (C) Harbin. (D) Wuchang. [file 12863_2021_995_MOESM4_ESM.docx]

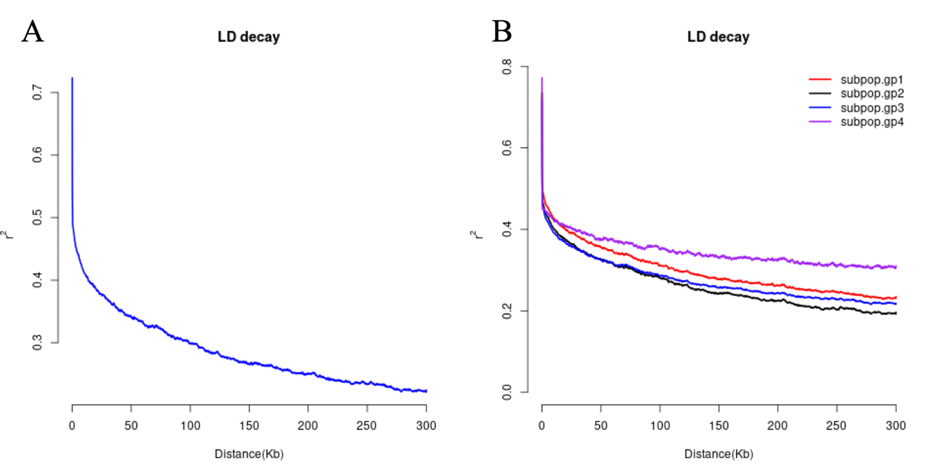


Supplementary Figure 1 (A) Linkage disequilibrium of all accessions across 12 chromosomes. (B) Linkage disequilibrium differences between four groups predicted by population structure analysis when K=4.


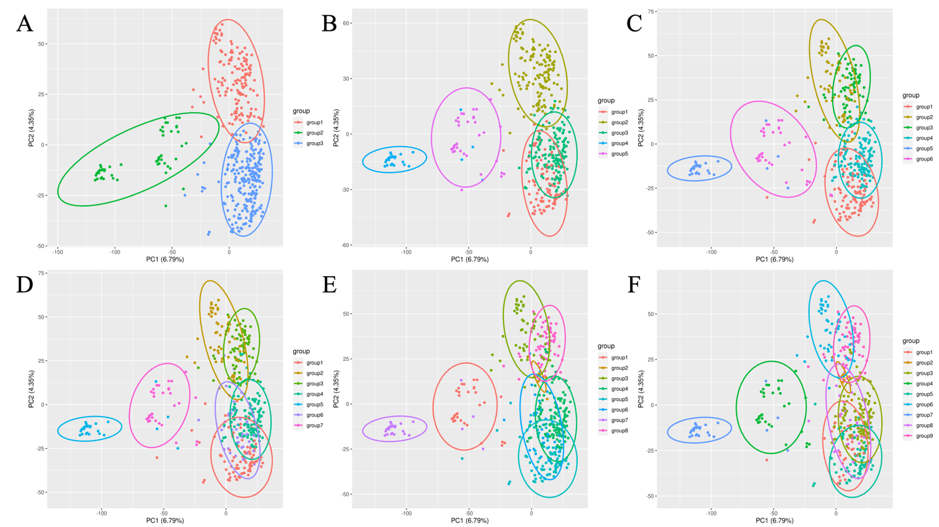


Supplementary Figure 2 Plots of the first two principal components in PCA with data points divided into 3 (A) and 5 to 9 (B-F) groups by faststructure.


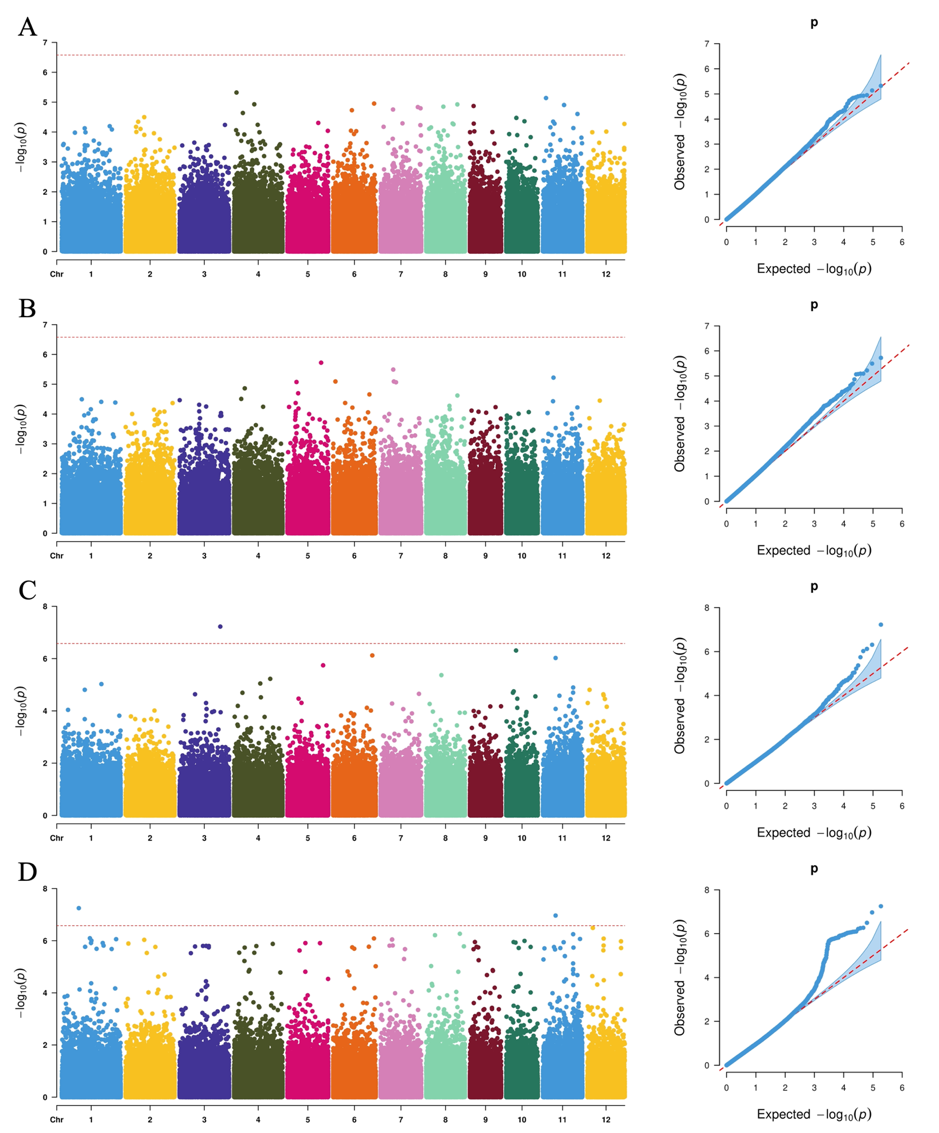


Supplementary Figure 3 Manhattan plots and QQ plots for the four traits in Harbin by MLM. (A) Days to heading. (B) Plant height. (C) Panicle weight. (D) Tiller number.


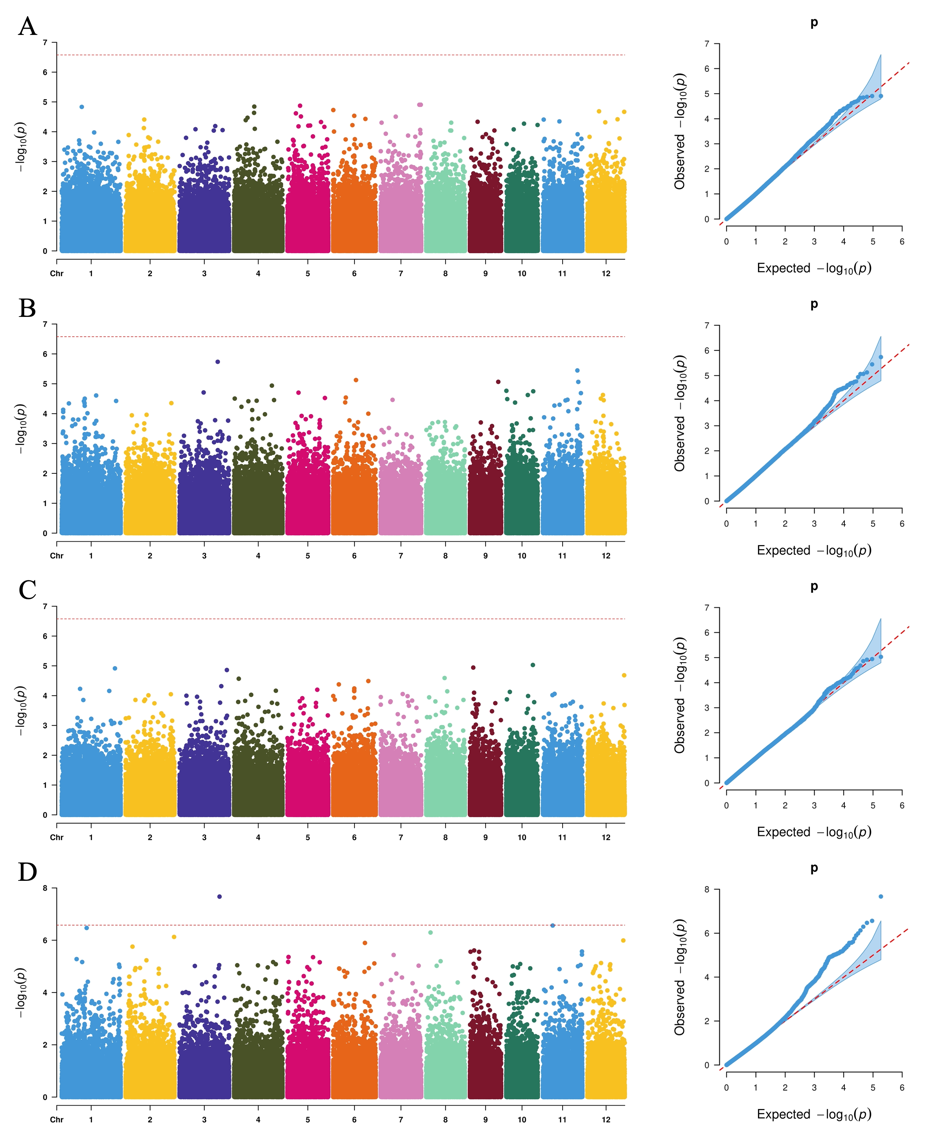


Supplementary Figure 4 Manhattan plots and QQ plots for the four traits in Jiamusi by MLM. (A) Days to heading. (B) Plant height. (C) Panicle weight. (D) Tiller number.


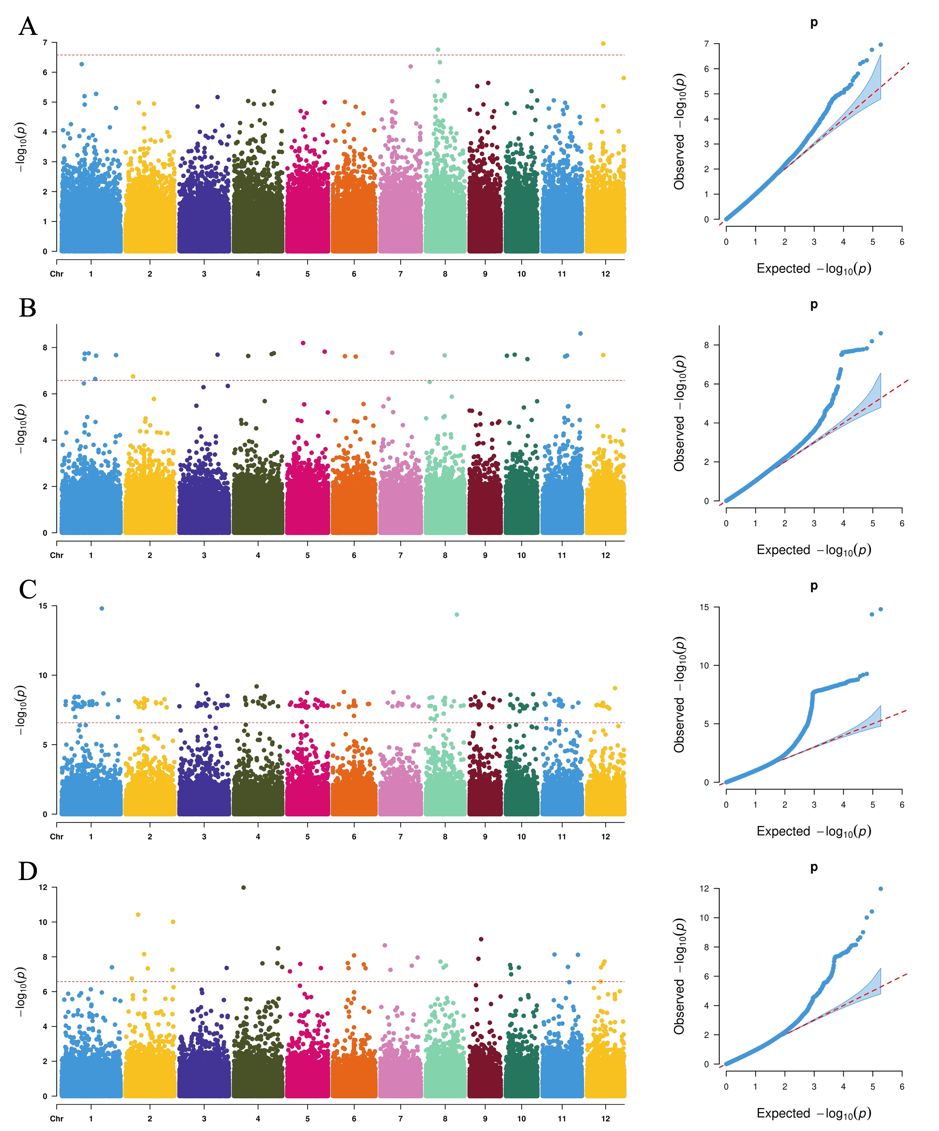


Supplementary Figure 5 Manhattan plots and QQ plots for the four traits in Wuchang by MLM. (A) Days to heading. (B) Plant height. (C) Panicle weight. (D) Tiller number.


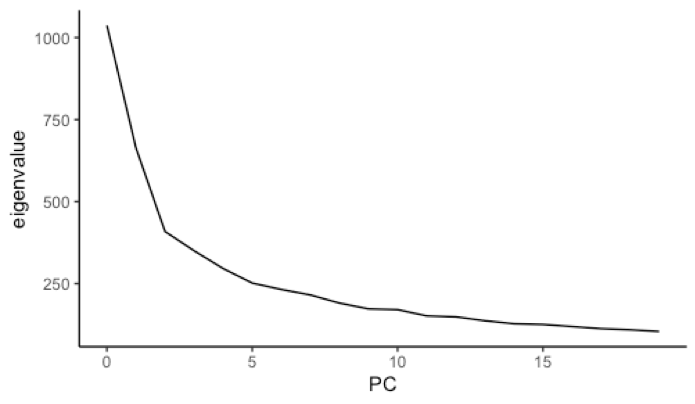


Supplementary Figure 6 Line plot of eigenvalues with the first 20 principal components. A significant downward trend was shown for the first 5 principal components.


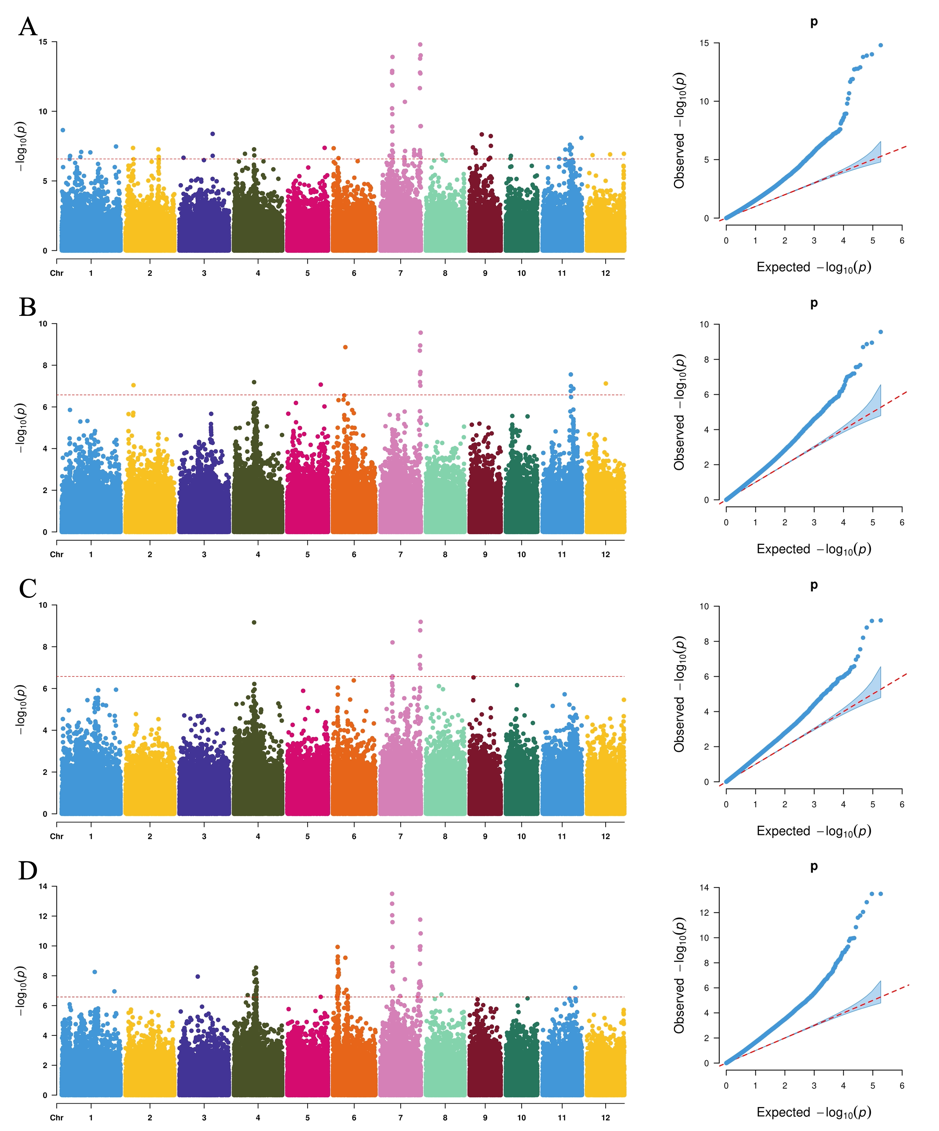


Supplementary Figure 7 Manhattan plots and QQ plots for Days to heading in four locations by GLM. (A) Heihe. (B) Jiamusi. (C) Harbin. (D) Wuchang.
